# A Treatise on the Principles and Practice of Medicine

**Published:** 1873-08

**Authors:** 


*A Treatise on the Principles and Practice of Medicine; Designed for the use of Students and Practitioners of Medicine.* By Austin Flint, M. D., Prof. of Principles and Practice of Medicine and of Clinical Medicine in Bellevue Hospital Medical College. Fourth Edition, Carefully Revised. Philadelphia: H. C. Lea, 1873. Buffalo: T. Butler & Son.

The excellent work of Dr. Flint is so widely known and prized, that anything more than a brief notice of the appearance of a fourth edition will be unnecessary. Embracing all departments of the Practice of Medicine Dr. Flint has in a style at once clear and condensed placed in the hands of practitioner and student a work which in many particulars is without a peer.

The five years which have elapsed since the publication of the third edition have witnessed many advances in the science of practical Medicine. To keep pace with this advance Dr. Flint has thoroughly revised the former edition making numerous corrections and additions. These additions are scattered through the whole work, the most noticable change however being in the department of Nervous Diseases. Dr. Flint's work will continue to merit the confidence of the profession, and the improvements made in the fourth edition will cause it more than ever to be regarded as the best American text book on Practical Medicine.

